# Beyond Glycemic Control: Biographical Disruption and the Lived Experience of Youth with Type 1 Diabetes

**DOI:** 10.2174/0115733998458230260223083418

**Published:** 2026-03-24

**Authors:** Ariela Orbea-Cevallos, Alejandra Aguirre-Benítez, Mayra Elizabeth Castillo-Gonzáles, Jaime David Acosta-España

**Affiliations:** 1 Health Sciences Faculty, Universidad Internacional SEK del Ecuador, Quito, Ecuador;; 2 College of Health Sciences, Universidad San Francisco de Quito, Quito, Ecuador;; 3 School of Medicine, Pontificia Universidad Católica del Ecuador, Quito, Ecuador;; 4 Centro de Investigación para la Salud en América Latina (CISeAL), Pontificia Universidad Católica del Ecuador, Quito, Ecuador

**Keywords:** Type 1 diabetes mellitus, adolescent health, biographical disruption, embodiment, lived experience, patient-centered care

## Abstract

**Introduction:**

Adolescents and young adults living with type 1 diabetes mellitus (T1DM) deal with distinctive challenges that go beyond clinical management. This review synthesizes qualitative evidence to examine how biographical disruption, embodiment, and illness narratives shape the lived experience of this population.

**Methods:**

Through a structured search across PubMed, Scopus, and Web of Science, 15 qualitative studies, published over the past decade, were identified. Thematic synthesis was conducted in seven domains: psychological burden, identity, adaptation, social experience, barriers, technology, and recommendations.

**Results:**

Emotional turbulence, anxiety, distress, and fear of hypoglycemia were common amongst adolescents with T1DM. Identity was often a major concern over biomedical risks, showing some gender differences in illness experience. Daily life involved vigilance, and attempts to regain normalcy were common in this population. In social matters, stigma, surveillance, and peer disconnection were prominent, but these concerns were ameliorated by family and friend support. Automated insulin delivery (AID) may cause reduction of burden but increased autonomy, though access was unequal due to economic, educational, and clinical barriers.

**Discussion:**

Findings emphasized how T1DM portrays disruption in biography, embodiment, and identity in adolescence and young adults, consistent with Charmaz’s framework of chronic illness. The absence of multidisciplinary approaches limits the detection of psychosocial needs and still frequently contributes to undesirable results. Culture, gender, and structural inequities shapes how young people experience, manage, and make sense of their condition.

**Conclusion:**

T1DM in youth is a lived reality that reshapes identity, autonomy, and social belonging. Handling biographical disruption requires culturally sensitive, developmentally adapted, and relationally grounded interventions. Future research needs to expand representation and adopt longitudinal studies of adaptation.

## 1 INTRODUCTION

Recent global estimates indicate a marked rise in Type 1 Diabetes (T1DM) prevalence, increasing by 13% from 8.4 million cases in 2021 to 9.5 million in 2025 [1], with the fastest growth occurring in low- and middle-income countries (LMICs), where the incidence of T1DM among children, adolescents, and young adults continues to rise [2]. These trends are consistent with projections by Gregory *et al.* [3], who anticipate up to 17.4 million global cases by 2040, and with those of Deng *et al.* [4], who attribute part of this increase to declining mortality rates, particularly in LMICs. T1DM is characterized by the autoimmune destruction of pancreatic β-cells, resulting in lifelong dependence on exogenous insulin [5, 6].

Adolescents and young adults with T1DM face unique medical challenges in managing their condition, and appropriate care requires high levels of self-management, patient- centered approaches, and multidisciplinary models [7-9].

Diagnosing T1DM in this population presents additional challenges in preventing both acute and long-term complications [10-12]. Acute events such as hypoglycemia and diabetic ketoacidosis (DKA) are commonly feared by patients and families [13]. Chronic complications such as retinopathy, peripheral neuropathy, arterial stiffness, and hypertension may affect one in three individuals with T1DM, with higher risks among youth from non-white ethnic backgrounds, those without health insurance, and those in low-income households [13-15].

Research has not equally captured these challenges across all age groups. Available evidence shows that pediatric T1DM research far exceeds that focused on adolescents and young adults. A comprehensive scientometric study documented substantial growth in pediatric research [16], contrasting with the limited adult epidemiological studies noted previously [17] and the lack of evidence-based clinical services and early-stage research on transition care for young adults highlighted by Monaghan *et al.* [18, 19].

Adolescence represents a transitional period in which individuals gain independence, but also struggle with new troubles [20, 21]. Transition from pediatric to adult healthcare is especially challenging for those with T1DM, as it may lead to reduced quality of care and poor glycemic control [13, 22]. During puberty, there might be physiological insulin resistance, generating a difficulty with glycemic regulation [3, 23]. For young people with chronic illnesses, age-related challenges can intersect with illness demands, which in turn, may entangle identity development [24, 25].

Chronic illness disrupts daily routines and knowledge systems [26, 27], resulting in experiences of fatigue, vulnerability, and body uncertainty [28], thus making pain, suffering, and mortality prominent worries and disturbing family systems and peer relationships.

Illness management uncertainties and social norms tend to influence relationships and life goals [29, 30]. These experiences can also be described as “disruptions in biography” and “disruptions of social relationships” [26]. These struggles highlight the need for care models that notice the lived experiences of youth with T1DM.

Bury’s concept of biographical and social disruption is consistent with the psychological and emotional burden of living with T1DM, further illustrating how chronic illness influences both identity and day-to-day management.

This review draws on Charmaz’s model of The Loss of Self and the Reconstruction of Self in Chronic Illness [31] to interpret lived experience in youth with T1DM. This model broadens Bury’s concept of biographical disruption by examining ways in which chronic conditions reshape identity, control, and social belonging. This framework enlightened the coding and thematic synthesis by pointing towards domains central to adolescence with T1DM-such as loss of control, identity renegotiation, social isolation, and experiences of disapproval [32-34]-thus providing a theoretical structure for the interpretation of qualitative findings.

T1DM causes a significant psychological burden on patients and their families [3, 35]. Diabetes-related distress encompasses the emotional stress of managing a chronic illness, which often overlaps with anxiety and depression in some patients [36, 37].

Diabetes may affect development, social relationships, and mental health due to its challenges [8, 38]. Physiological and psychosocial changes during adolescence can exacerbate these difficulties, resulting in poor adherence, risky behaviours, and emerging eating or mental health issues that can further complicate disease management [8, 39]. Despite the existence of validated tools such as the T1-DDS, their use among adolescents remains inconsistent [40].

Adolescents and young adults with T1DM can also face increased anxiety, negative self-image, and constant emotional challenges, compared to their healthy peers [41, 42]. Diabulimia, the intentional omission or manipulation of insulin for weight control, still remains poorly defined [43, 44]. Even though estimates suggest that up to 30% of adolescents and young adults with T1DM use insulin omission as a compensatory behavior, the condition is often underdiagnosed and undertreated [36, 45, 46].

Latest trials with advanced automated insulin delivery (AID) systems have shown improved effectiveness in HbA1c level control [47-49]. Other evidence indicates reductions both in hypoglycemia and hyperglycemia duration and decreased distress, thus less risk of DKA or severe complications [50]. Despite the evidence, interventions remain primarily biomedical and reflect the need for empathy, narrative skills, and comprehensive psychosocial care.

George Engel [51] first proposed The Need for a New Medical Model, which introduces a biopsychosocial perspective that continues to influence modern healthcare. In accordance, medical practice has recently paid attention to prioritizing patient involvement in decision-making; shifting from physician-centered discussions to collaborative care that acknowledges patients as active participants [52, 53]. However, healthcare professionals have the challenge to recognize patients’ struggles, show empathy, and genuinely engage with patient experiences [54, 55]. Healthcare professionals must have the ability to interpret and guide illness narratives that help view disease management and personal identity.

Nonetheless, meta-analyses show healthcare efforts continue to focus on adherence, glycemic control, and preventing complications [56-58], overlooking emotional support. Resulting in concepts like biographical disruption, lived experience, and embodiment being underrepresented in research and healthcare practice.

Previous reviews have noted the emotional burden of T1DM in adolescents and young adults [59, 60], but a preventive approach centered on mental health is lacking. This review intends to fill that gap by analyzing qualitative studies on the lived experiences of adolescents and young adults with T1DM, highlighting biographical disruption, embodiment, and illness narratives to further understand identity, relationships, and transitions. Leading to uncover opportunities for clinical practice and future research.

## 2 MATERIALS AND METHODS

### 2.1 Study Design and Objective

This review explores the lived experiences of adolescents and young adults with T1DM, focusing particularly on biographical disruption, embodiment, and illness narratives. The review aims to note, evaluate, and synthesize original qualitative research on how this population understands their illness in regards to their identity, social relationships, and life changes.

### 2.2 Search Strategy

A bibliographic search was independently conducted by two reviewers (AOC and AAB) on August 1, 2025, across three databases: PubMed (n=34), Scopus (n=28), and Web of Science (n=27). The search combined free-text terms using Boolean operators to identify studies exploring the experiential aspects of T1DM in youth.

The following database-specific search strings were applied:

**PubMed:** (“biographical disruption” OR “embodiment” OR “illness narrative” OR “lived experience”) AND (“diabetes mellitus type 1” OR “T1DM”) AND (adolescent OR youth OR “young adult”)

**Scopus:** TITLE-ABS-KEY (“diabetes mellitus type 1” AND (“biographical disruption” OR embodiment OR “lived experience” OR “illness narrative”) AND (adolescent OR “young adult” OR youth))

**Web of Science:** TS=((“type 1 diabetes” OR T1DM) AND (“biographical disruption” OR embodiment OR “lived experience” OR “illness narrative”) AND (adolescent OR youth OR “young adult”)).

During the initial search, no filters were applied to maximize sensitivity. The search was restricted to articles published in English or Spanish within the last 10 years (August 2015-August 2025).

### 2.3 Eligibility Criteria

Studies included met the following criteria: Peer-reviewed original research. Published in English or Spanish. Focused on adolescents or young adults (ages 10-25) diagnosed with T1DM. Used a qualitative or mixed-methods design. Disclosed experiential outcomes in relation to illness perception, identity, or psychosocial impact.

**Exclusion criteria were:**

Studies focused on other types of diabetes (*e.g.*, type 2, gestational)

Samples composed exclusively of children (<10 years) or older adults (>25 years)

Publication types such as reviews, editorials, letters, and case reports

Studies lacking relevant experiential or narrative outcomes

### 2.4 Screening and Selection Process

The initial search found 89 articles. After removing duplicates, 40 unique articles remained.

Titles and abstracts were independently screened by both reviewers using predefined relevance criteria. To strengthen transparency, inter-rater agreement was calculated during the title/abstract screening stage (Cohen’s κ = 0.78), indicating substantial agreement. Screening was conducted separately by two reviewers (AOC and AAB). Any initial disagreements were discussed until consensus, following established procedures for qualitative synthesis. Of the 40 records:

17 were excluded for lack of relevance to the research question

6 did not align with the study aim (*e.g.*, focused only on biomedical outcomes)

2 were inaccessible in full text

A final sample of 15 articles was included for full-text analysis.

### 2.5 Data Extraction and Synthesis

Data was gathered using a standardized template in Microsoft Excel 365 (2025 version) to record details such as author, year, country, study design, sample characteristics, data collection method, analytical approach, and key experiential findings.

Bury´s biographical disruption, embodiment, and Charmaz’s Loss and Reconstruction of Self were used as reference concepts that informed the development of initial codes and guided the organization of themes. Later, these findings were organized into conceptual domains, which included identity formation, social relationships, and life transitions.

### 2.6 Quality Appraisal

The approach in this review aligns with current methodological guidance. Methodological quality and rigor were independently assessed by both reviewers using the Critical Appraisal Skills Programme (CASP) (2024) [61]. Each study was evaluated across 10 domains, including clarity of aims, appropriateness of design, recruitment, data collection, reflexivity, ethics, and analytical rigor.

CASP was used for interpretation rather than as an exclusion tool; studies with higher CASP scores contributed more strongly to thematic depth. This approach aligns with recent methodological reflections, highlighting the value of interpretive use of quality appraisal in qualitative synthesis [62].

### 2.7 Reporting Standards

This review follows the ENTREQ (Enhancing Transparency in Reporting the Synthesis of Qualitative Research) guidelines.

## 3 RESULTS

The initial search found 89 records; after removing the duplicates, 40 articles were selected and were independently screened by two reviewers. After excluding articles for irrelevance (n=17), inconsistent with the study aim (n=6), and lack of access (n=2), a final sample of 15 articles was included for full-text analysis. Supplementary Table **1** has the complete data summary of the results of each manuscript.

### 3.1 Psychological and Emotional Burden

T1DM represents an emotional challenge and has its psychological complexity, specifically for adolescents and young adults [63]. The chronicity of the disease and its emotional demands results with participants reporting an ongoing worry about daily management and long-term complications [64, 65]. Treatment fatigue and adherence produce emotional fluctuations [66], while hypoglycemia commonly generates fear [64].

Severe problems regarding mental health in several context settings include distress, anxiety, suicidal thoughts, and poor sleep in this population [67]. Transitioning to adulthood is a sensitive period that needs technical skills and psychological adjustment since roles and responsibilities change during this time [27]. This experience demonstrates how constant vigilance, somatic uncertainty, and fear associated with T1DM generate a biographical disruption.

### 3.2 Self-perception and Identity

Issues about identity formation are frequent; adolescents and young adults often prioritize their self-concept over associated biomedical risks [27]. T1DM is characterized by disrupting the body, emotionally drain, and increase awareness of mortality [67]. How these adolescents and young adults integrate their identity is different; some participants include T1DM as part of their self-concept, and others see it as an external facet of their identity [58].

Gender differences are contemplated; men tend to compartmentalize the disease, and women are more likely to share their experience [58]. However, gender does not differ in the development of self-management needed to adapt to a changed life course [59]. T1DM is illustrated as a disruptive force that impacts self-continuity, and individuals work on strategies to maintain social ties [27]. Together, these identity issues demonstrate how T1DM needs an ongoing reconstruction of the self, as stated by Charmaz.

### 3.3 Adaptation and Daily Living

Day to day, adolescents and young adults with T1DM have to be constantly vigilant to self-monitor and ready to handle unpredictability [68]. Participants report their routines shaped by the disease, with diagnosis marking major life changes and an inevitable shift in their lives [69].

Over time, many participants describe developing a sense of agency and acceptance that contributes to the integration of diabetes management [66]. Some positive aspects are reported, such as healthy eating, physical activity, and participation in diabetes camps or fundraising, which are recognized after the adaptation process [70]. These findings show how they gradually reconstruct a sense of normalcy by developing new embodied routines and interpretations of the illness.

### 3.4 Social Experience and Support

Social experiences in adolescents and young adults with T1DM can be characterized by both supportive and stigmatizing interactions. Commonly, they find it difficult to place their illness within larger social contexts, resulting in feelings of isolation [27]. T1DM causes a differentiation from peers [27, 63, 64, 70]. Participants report that family, friends, and healthcare providers often apply “tough love” [71]. Misconceptions from laypeople are common and foster a constant pressure to portray perfect control to avoid being called a “bad diabetic” [71].

Myths such as the confusion between T1DM and type 2 diabetes generate negative social effects like discomfort or negative reactions in adolescents and young adults since they are sensitive to health moralism, where their lifestyle choices are judged [68]. According to Ketema Bogale *et al.* [65], these interactions shape relationships and participation in social contexts. Communicating their condition is common but done with fear of stigma and frustration [70], as well as administering insulin in public [27].

Social support is extremely important in managing diabetes. Adolescents who share their diagnosis with peers often report better treatment adherence [65]. Most adolescents in an article reported being content to share their diagnosis with friends, since this encourages them to adhere to their treatment plan [63]. Adolescents and young adults recognize that school contexts without that social support can be stressful [69]. Social support also helps with balance and incorporation of diabetes management into day-to-day [61]. These narratives reveal how biographical disruption extends to social relationships, shaping social life.

### 3.5 Barriers

Additionally, there are barriers affecting adolescents and young people living with T1DM. Jocic *et al.* [71] highlighted limited access to medical devices related to the disease and institutional definitions of disability. Also, Ketema Bogale *et al.* [65] described economic issues as important, such as concerns regarding obtaining supplies and complications related to lower socioeconomic status and structural inequities [67].

In the educational environment, some interferences include fewer contact hours, school dropout, narrow concentration, and interrupted academic progress [72]. Some adolescents report that schools can lack support, specifying limited understanding from educators [69].

Healthcare systems are also viewed as obstacles to autonomy and quality of life. Many young people reported that the medical system itself hindered their ability to lead a normal life [27]. Concerns were also raised about not being treated as individuals by healthcare professionals, as well as the tendency for providers to involve parents more than the adolescents and young people themselves [64]. These structural and interpersonal barriers intensified the biographical and embodied disruption of T1DM by limiting autonomy and constraining the ways young people could interpret, express, and manage their illness.

### 3.6 Use of Automated Insulin Delivery

Three studies examine the impact of AID systems in adolescents and young adults with T1DM, reporting reductions in cognitive, emotional, and relational burden, less time spent on management tasks, and greater flexibility in daily routines [73]. Rankin *et al.* [74] additionally describe improved normalization in social settings, reduced family conflict, and fewer worries about hypoglycemia, though some adolescents reported hiding equipment due to self-consciousness.

AID systems supported both those who preferred active involvement and those seeking distance from the condition, and participants reported increased independence, reduced anxiety, and better sleep quality [74-75]. Across studies, AID technologies helped reduce biographical disruption by easing cognitive and emotional burden, even as their visibility maintained the embodied presence of the illness.

### 3.7 Recommendations

Two studies offered recommendations to improve the lived experience of adolescents and young people with T1DM. Schønning *et al.* [76] reported that using existing technological tools aids in monitoring and managing diabetes, promotes independence in care, and enhances young adults’ sense of autonomy. They also discussed the importance of narrative work by healthcare professionals, where clinical interactions go beyond biomedical factors, and noted that setting realistic standards for diabetes care helps build trust and supports personalized care. Shattnawi and Mahassneh [66] pointed out adolescents’ views, such as the need to increase knowledge and awareness, expand resources, and encourage social integration. Additionally, their findings emphasize that social interactions and a sense of belonging are equally relevant as material resources in managing the illness. These recommendations highlight the need for healthcare approaches that recognize identity reconstruction and acknowledge the embodied disruptions that adolescents and young adults with T1DM experience.

## 4 DISCUSSION

Under biomedical models, exclusively medical challenges were considered while living with T1DM during adolescence and young adulthood. This disease notably disrupts personal history, physical experience, and sense of self. This review merges qualitative data across seven areas, demonstrating how a chronic illness has an impact not only on health outcomes but also on the development of identity and social connection. These results amplify earlier reviews that report the psychological impact of T1DM in youth and their families [59, 60] by demonstrating how the emotional process shapes identity, embodiment, and social belonging. While previous research centers on anxiety, depression, and family distress [59] or wider psychological issues [60], the results of this review elucidate the relational, narrative, and symbolic ways adolescents and young adults experience their disease.

Articles describe emotional turbulence closely related to embodied experience, for instance, bodily changes, constant monitoring, and consternation of unpredictability related to hypoglycemia or long-term complications. This predictability strengthens chronic illness as a process in which the body undergoes disruption, additionally from the difficulty of cognitive interpretation and the need for psychosocial support.

Identity negotiation arose as a dynamic, relational, and embodied process. Adolescents reconstructed a sense of self in the context of the demands of bodily vigilance, volatile symptoms, and social recognition or misrecognition from people in their social networks [27, 58, 63]. Gender differences in narrative integration [63] indicate that identity is shaped not only by coping strategies but also by cultural expectations. Social relationships-whether supportive or stigmatizing-are relevant to understand if biographical disruption was either exacerbated or restored.

Charmaz’s model of The Loss of Self and the Reconstruction of Self in Chronic Illness (1983) was selected since it elaborates on Bury’s concept of biographical disruption and, through grounded theory, analyzes psychosocial processes that overlap across different chronic illnesses [32]. Her model aligns closely with the identity and social topics seen in T1DM. Charmaz [34] postulates that chronic illness enforces limits on daily life (1995), commonly leading to a perceived loss of control, specifically related to acute complications [31] and treatment fatigue with difficulty of adherence [61]. Additionally, impacting identity, as the lived experience of T1DM not only physically disrupts and emotionally drains [33], but also embodies constant self-monitoring and variation of symptoms [28], and adopting various patterns of identity integration [63].

Social isolation, another cornerstone of Charmaz’s model (2005), was solidly evident in this review, in which the targeted population reported feeling different from their peers [27, 63, 64, 70]. Charmaz [33] also indicates a recurring experience of discreditation (2020), noticeable in results related to stigma, myths, and pressure to avoid being labeled [68, 71, 70]. Charmaz's concept of “becoming a burden” (2005) was not distinguished in this review, probably because of the specific population of the included articles. Altogether, Charmaz’s framework can be explicative of how T1DM disrupts control, identity, and social belonging during adolescence and young adulthood.

Technological innovations, such as AID, were associated with decreasing burden and increasing autonomy [73, 74], aligning with findings from the Bionic Pancreas Research Group (2022) [47] and Godoi *et al.* [50]. These findings indicate that AID is not only a medical improvement but a mechanism of biographical repair.

There are some structural barriers that include-limited availability of resources [71], economic inequalities [65], and school-based exclusion [72]-that limit access to the possibility of biographical repair, underlining how embodiment is shaped not only by the illness but by healthcare and socioeconomic context. Besides technological opportunities, an article emphasizes the importance of narrative abilities of healthcare providers and promotion of social awareness and inclusion [66], reinforcing the integration of narrative medicine into medical education [77]. These approaches seek identity-centered care that considers self-image and relationships in addition to glycemic management. Therefore, interventions need to adapt to developmental stages, cultural backgrounds, and relational dynamics [78, 79].

The findings of this review have significant implications for healthcare providers, educators, and policymakers. For the medical field, adolescents’ reports of feeling misunderstood highlight the need for narrative abilities and communication training that acknowledges the loss of control and identity disruption stated by Charmaz’s framework.

In areas of mental health, professionals must address the emotional distress, anxiety, identity conflicts, and possible suicidal ideation, with a focus on age and gender. Educators also play an important role by fostering inclusive school contexts that lessen stigma and normalize illness in these stages of life.

Policymakers must handle structural inequities indicated—such as economic barriers and unequal access to innovation devices for diabetes management—to ensure healthcare models that promote adolescents and young adults to be more autonomous. Overall, these recommendations spotlight the importance of systems that respond to the lived experiences of adolescents and young adults with T1DM rather than focusing only on biomedical solutions.

## 5 LIMITATIONS

This review has several limitations. First, the synthesis included only qualitative studies published in English or Spanish, which may exclude valuable perspectives from other cultures and languages. Second, although a structured search strategy was applied, inconsistent indexing of qualitative research may have resulted in missed articles. Third, despite the use of CASP appraisal, variability in methodological reporting across selected studies may have influenced the depth and balance of the synthesis. Fourth, most included studies came from high-income countries, limiting the applicability of findings to LMICs. Finally, the review consisted of an interpretation using Charmaz’s framework and therefore provides conceptual insight rather than generalization.

## CONCLUSION

Adolescents and young adults with T1DM undergo disruptions to their day-to-day lives that impact their identity, autonomy, and social interactions. Throughout the included articles, participants described emotional stress, continuous self-monitoring of bodily changes, and difficulties incorporating T1DM into their identity. Technological innovations such as AID were found to be beneficial, although results also indicate that unequal access exists and user experiences are mixed. These findings highlight the need for care strategies that address not only metabolic goals but also the psychosocial aspects revealed in the data. Future research should include representation from under-researched countries and explore how these stages of life adapt to T1DM.

## LIST OF ABBREVIATIONS

AID: Automated Insulin Delivery
DKA: Diabetic Ketoacidosis
T1DM: Type 1 Diabetes Mellitus
LMICs: Low- and Middle-income Countries
