# Supplementary material for: Beyond Glycemic Control: Biographical Disruption and the Lived Experience of Youth with Type 1 Diabetes
Source: Curr Diabetes Rev. 2026 Mar 24;22(10):E15733998458230. doi: 10.2174/0115733998458230260223083418 (PMC13725147; doi:10.2174/0115733998458230260223083418)
Supplement: Supplementary file 1 [file CDR-22-10-E15733998458230_SD1.pdf]

Supplementary Material

Beyond Glycemic Control: Biographical Disruption and the Lived Experience of Youth with Type 1 Diabetes

Ariela Orbea-Cevallos[1,*] , Alejandra Aguirre-Benítez[2] , Mayra Elizabeth Castillo-Gonzáles[1] and Jaime David Acosta-España[1,3,4]

[1]Health Sciences Faculty, Universidad Internacional SEK del Ecuador, Quito, Ecuador; [2]College of Health Sciences, Universidad San Francisco de Quito, Quito, Ecuador; [3]School of Medicine, Pontificia Universidad Católica del Ecuador, Quito, Ecuador; [4]Centro de Investigación para la Salud en América Latina (CISeAL), Pontificia Universidad Católica del Ecuador, Quito, Ecuador

Table S1. Summary of findings for included studies.

Title	Reference	Country	Participants (age, N)	Data Collection	Results
Developing a personal and social identity with type 1 diabetes during adolescence: A hypothesis generative study	Commissariat et al. [63] (2016)	USA	Adolescents, 16 ± 1.9, N=40	Guided interviews	Perception of diabetes as a burden; identity negotiation; difference from peers; gender differences in communication.
Parent, child, and adolescent lived experience using the insulin-only iLet Bionic Pancreas.	Howard et al. [73] (2024)	USA	Children, adolescents, parents, N=52	Focus groups (UTAUT framework)	Less burden, more freedom, optimized routines, better daily management, and decreased stress with technology use.
The lived experience of young adults emotionally labouring with type 1 diabetes: Findings of an interpretive phenomenological study	Jocic et al. [71] (2024)	Online	Young adults 18–30, N=9	Semi-structured interviews (IPA)	Themes: tough love, external misconceptions, systemic barriers; intrapersonal struggles.

Lived Experience of People with Type 1 Diabetes in North East Ethiopia: Psycho-Social and Economical Perspective, a Phenomenological Study	Ketema Bogale et al. [65] (2023)	Ethiopia	Adolescents/young adults 14–26, N=9 (total 13)	Semi-structured interviews	Domains: economic (stress related to various supplies), psychological (coping mechanisms), social (isolation, stigma, support system).
Perceptions of Adolescent Patients of the “Lived Experience” of Type 1 Diabetes	King et al. [64] (2017)	UK	Adolescents 16–21, N=20	Semi-structured interviews	Adjustment includes developing skills, regulating emotions, and communication concerning peer differences.
Drawing and Dialogue: Youth’s Experiences With the “Face” of Diabetes	Liesch & Elertson [71] (2020)	USA	Children 8–15, N=20	Interviews + drawings	Positive aspects include a healthier lifestyle, diabetes camps, and fundraising; challenges such as feeling different, stigma, frustration, and worries about the future.
The lived experience of young Australian adults with type 1 diabetes	Mullan et al. (2020) [68]	Australia	Young adults 18–35, N=25	Semi-structured interviews	Day-to-day vigilance; negative social reactions, myths about T1DM and T2D, and lifestyle judgment involving health moralism.
Living with type 1 diabetes and schooling among young people in Ghana: a truism of health selection, inadequate support, or artefactual explanation of educational inequalities?	Owusu & Doku [71] (2024)	Ghana	Adolescents/young adults 14–24, N=28 + caregivers	Semi-structured interviews	Educational disruption: absenteeism, dropout, reduced concentration, altered school choice; coping shaped by structural constraints.

A<br><br>A qualitative study of type 1 diabetes complications, mental health, and structural pathways of complications occurrence among young people (14–24 years) and caregivers in southern Ghana.	Owusu et al. [67] (2024)	Ghana	Adolescents/young adults 14–24, N=28 + caregivers & providers	In-depth interviews	Complications (visual impairment, neuropathy, reproductive health); psychological distress (anxiety, suicidality); socioeconomic and structural barriers linked to poor outcomes.
Adolescents' and their parents' experiences of using a closed-loop system to manage type 1 diabetes in everyday life: qualitative study.	Rankin et al. [74] (2022)	UK	Adolescents 10–17, N=18 + parents	Semi-structured interviews	Closed-loop systems facilitated normalization, reduced hypoglycemia concerns, decreased parental prompting; some concealment to avoid stigma.
The lived experiences of young people living with type 1 diabetes: A hermeneutic study.	Rising Holmström & Söderberg [69] (2022)	Sweden	Adolescents 13–18, N=10	In-depth interviews	Diagnosis experienced as irreversible change; family burden; centrality of body; lack of school support; disruption of familiar world.
Hybrid closed- loop therapy with a first-generation system increases confidence and independence in diabetes management in youth with type 1 diabetes.	Roberts et al. [75] (2022)	Australia	Adolescents/young adults 12–25, N=17 + parents	Semi-structured interviews	Hybrid closed-loop increased independence, reduced anxiety, and improved sleep.

Disruptive illness contexts and liminality in the accounts of young people with type 1 diabetes.	Sanders et al. [27] (2019)	UK	Young adults 16–24, N=32 (FG) + 15 interviews	Focus groups + interviews	Identity challenges, stigma in public self-management, tension between social life and control, frustration with the medical system, and biographical disruption in public, clinical, and knowledge contexts.
From Guidelines to Lifelines—An Ethnographic Study of How Diabetes Management Is Emplotted During Clinical Encounters with Young Adults with Type 1 Diabetes.	Schønning et al. [76] (2025)	Denmark	Young adults 18–23, N=14	Ethnographic observation + conversations	Narrative work in healthcare promotes autonomy, trust, individualized care, and technology as a tool for constructing hopeful futures.
Balancing life with type 1 diabetes mellitus: A qualitative study of Jordanian adolescents.	Shattnawi & Mahassneh [66] (2025)	Jordan	Adolescents 16–19, N=10	Semi-structured interviews	Themes: constant struggle (emotional/social challenges); coping via social support and integration; search for agency, normalcy, and improved environment/resources.
